# Experience with mental illness and awareness of health or crisis services among adults in Houston, 2024–2026

**DOI:** 10.3389/fpubh.2026.1900430

**Published:** 2026-10-28

**Authors:** Emmanuel C. Okolo, Amber Kearney, Gary Wingenbach, Josephine Engels, Andrea Usanga

**Affiliations:** 1 Department of Agricultural Leadership, Education, and Communications, Texas A&M University, College Station, TX, United States; 2 Mental Health America of Greater Houston, Houston, TX, United States

**Keywords:** awareness, healthcare access, mental health, mental illness, sociodemographic

## Abstract

Mental healthcare needs are growing, yet awareness of available services remains limited, even in Houston, Texas. This study examined relationships between having experienced or been diagnosed with mental illness and awareness of community-based mental health or crisis services. Approximately 1,000 Houston-area adults were surveyed in 2024 and 2026. Chi-square and multivariable regression analyses showed that experience with mental illness was associated with SGM populations, race/ethnicity, and age after adjustment for sociodemographic characteristics. SGM participants had greater odds of reporting mental illness, whereas Black/African American, Hispanic/Latinx, Asian, and older adults had lower adjusted odds relative to their reference groups. Awareness of community-based services was independently associated with gender, SGM populations, race/ethnicity, age, and annual household income; females had greater odds of awareness, whereas Hispanic/Latinx, Asian, and older adults had lower odds. Findings highlight sociodemographic differences in reported experience with mental illness and awareness of community-based services. Longitudinal research is needed to further examine these relationships.

## Introduction

Mental health is a significant concern in the United States, impacting nearly 20% of adults annually and almost 50% over their lifetime (1). Annually, ~16% of youth aged 6 to 17 and over 33% of young adult’s aged 18 to 25 exhibit symptoms, underscoring their susceptibility (2). In both Europe and the United States, between 52 and 74% of individuals suffering from mental illness do not receive adequate care (3), and worldwide, only 42 to 44% seek treatment (4). The situation in Texas remains critical; despite the widespread mental health needs, awareness of available resources remains low, especially in rural and low-income populations (1).

Sociodemographic factors are widely recognized as risk factors for mental health issues and service use (4). A systematic review found weak evidence linking demographics—age, gender, ethnicity, educational level, and marital status—to service utilization (3–5); no association was found between income and mental health service use. While sexual and gender minority (SGM) populations had greater risks of experiencing depression, anxiety, suicidality, and substance abuse, few studies have explored whether SGM is related to mental healthcare use (6).

Despite widespread availability of mental health education and intervention programs, evidence suggests their effectiveness in increasing awareness, especially for disadvantaged and vulnerable populations, is limited, leading to untreated mental health illnesses (7, 8). The present study examined associations between having experienced mental illness, awareness of community-based mental health or crisis services, and/or selected sociodemographic factors.

### Purpose and questions

The purpose of this study was to determine if relationships existed between mental illness and awareness of community-based mental health or crisis services among Houston adults from 2024 to 2026. Two questions guided this inquiry.

1. Is there a significant relationship between having experienced or been diagnosed with mental illness and awareness of community-based mental health or crisis services?
2. Do relationships exist between mental illness, awareness of services, and/or sociodemographic characteristics?

## Methods

A comprehensive understanding of the mental health landscape and mental healthcare services promoted through Mental Health America of Greater Houston (MHAGH) in Harris and Fort Bend Counties, Texas, was conducted among adults from diverse demographic and community backgrounds. This study used a quantitative, descriptive-correlational design grounded in a postpositivist worldview to examine relationships among key variables without manipulating them (9).

The first sample (*N* = 500) of participants were from a non-probability, opt-in panel administered by the survey distribution partner, Prodege, in mid-July 2024. Participants may have received incentives from the survey distribution partner, although the exact incentive amount or type is unknown. The second sample (*N* = 500) was also recruited using a non-probability, opt-in approach through Marketing Systems Group in mid-January 2026. The samples were pooled for analysis.

The survey instrument was designed to assess the prevalence of mental health concerns, coping strategies, awareness, and use of available resources, and perceptions of mental healthcare in the Houston area. Eighteen closed- and open-ended questions measured the variables of interest; there were 10 demographic questions. Closed-ended questions used scales (1–10; 1–5), single-choice options (Yes/No), and ranked items to answer the questions. Example questions included “What kinds of mental health or crisis services are you aware of in your community?” (Open-ended) and “Are you aware of the mental health or crisis services that are available in your community?” (Yes/No). All measures were self-reported.

Data were analyzed using descriptive and inferential statistics. Chi-square tests of independence examined bivariate associations between the study outcomes and sociodemographic characteristics, with Cramér’s *V* used to estimate effect size. Multivariable regression models were then used to assess adjusted associations while controlling simultaneously for gender, SGM populations, race/ethnicity, age, educational level, marital status, annual household income, and number of children under 18 in household. Because sparse observations in the “Not Sure” category of the mental illness item produced unstable multinomial estimates, its adjusted analysis was restricted to definitive Yes/No responses and estimated using binary logistic regression. Awareness of community-based mental health or crisis services was analyzed using multinomial logistic regression, with “No” as the reference outcome. Adjusted odds ratios (AORs), 95% confidence intervals (CIs), and likelihood-ratio tests were reported. Statistical significance was established at *p* < 0.05.

## Results

Participants (*N* = 1,003) are described in Table 1. Most were female (55%), identifying as heterosexual (90%), 25–44 years old (41%), and White (38%). The largest proportion had some college or a two-year degree (30%), although the largest income category was $50,000–$74,999 (22%). Most (47%) reported being single, and more than half (57%) did not have any children under 18 in their household.

**Table 1:** Descriptive statistics of study participants (*N* = 1,003).

Variables	Categories	*f*	%
Gender	Female	551	54.9
	Male	450	44.9
Sexual orientation	Heterosexual	900	89.7
	SGM populations	85	8.5
Age	18–24	122	12.2
	25–44	412	41.1
	45–64	314	31.3
	65+	154	15.4
Race/ethnicity	Black/African American	234	23.3
	White	383	38.2
	Hispanic/Latinx	276	27.5
	Asian	85	8.5
	Indigenous/Others	25	2.5
Education level	Less than high school	33	3.3
	High school graduate	244	24.3
	Some college or associate degree	304	30.3
	College graduate	289	28.8
	Post-graduate	126	12.6
Annual household income	< $30,000	219	21.8
	$30,000–$49,999	190	18.9
	$50,000–$74,999	225	22.4
	$75,000–$99,999	117	11.7
	$100,000–$149,999	132	13.2
	$150,000+	83	8.3
Marital status	Single	470	46.9
	Married	404	40.3
	Domestic partnership	74	7.4
	Others/Unknown	45	4.5
Number of children under 18 in household	0	569	56.7
	1–2	346	34.5
	3–6+	88	8.8

Responses may not equal 100% because some chose to opt out of answering some items.

The first question was to determine if a significant relationship existed between having experienced or been diagnosed with mental illness and awareness of community-based mental health or crisis services. A Chi-square test of independence (Table 2) produced a statistically significant relationship between the variables, *χ*^2^ (4, *N* = 998) = 26.25, *p* < 0.001, Cramér’s *V* = 0.12. Individuals who experienced or were diagnosed with mental illness were more aware than others of the community-based mental health or crisis services.

**Table 2:** Chi-square test summary (*N* = 998).

Test	Value	df	*p*-value
Chi-square (*χ*^2^)	26.25	4	<0.001
Cramér’s *V*	0.12		

***p < 0.001.

Table 3 presents the cross-tabulation results for awareness of mental health services across levels of experienced mental illness. Among participants who reported no mental illness, most 35.36% were unsure of their awareness of mental health services. Participants who were unsure whether they had experienced mental illness most frequently reported no awareness of mental health services (40.43%). However, the strongest awareness of mental health services came from respondents (49.10%) who reported having experienced mental illness. These patterns suggest those with lived experiences of mental illness had greater awareness of available mental health or crisis services in their community.

**Table 3:** Crosstab distribution of mental health experience and awareness of mental health services (*N* = 998).

Experienced mental illness	Awareness of mental health services					
	No	%	Unsure	%	Yes	%
No	167	29.82	198	35.36	195	34.82
Not sure	19	40.43	17	36.17	11	23.40
Yes	88	22.51	111	28.39	192	49.10

Values represent the observed counts with row percentages.

Chi-square analyses in Table 4 revealed that mental illness experience was significantly associated with SGM populations, race/ethnicity, age, and marital status, whereas gender, educational level, annual household income, and number of children under 18 in household were not significantly associated with mental illness experience. Among the significant associations, SGM populations (Cramér’s *V* = 0.177) and age (Cramér’s *V* = 0.168) showed the comparatively largest effect size, although associations remained small. Awareness of community-based mental health or crisis services was significantly associated with gender, SGM populations, race/ethnicity, age, educational level, and annual household income, while marital status and number of children under 18 in household were not significantly associated with awareness. Race/ethnicity (Cramér’s *V* = 0.135), gender (Cramér’s *V* = 0.119), and annual household income (Cramér’s *V* = 0.116) showed the comparatively largest associations with service awareness. Collectively, the findings showed a small effect size, with Cramér’s *V* values ranging from 0.027 to 0.177.

**Table 4:** Chi-square tests of associations between sociodemographic characteristics and study outcomes.

Demographic variable	Experienced mental illness					Awareness of mental health services				
	*N*	*χ^2^*	df	*p*	Cramér’s *V*	*N*	*χ^2^*	df	*p*	Cramér’s *V*
Gender	999	4.16	2	0.125	0.065	998	14.20	2	< 0.001	0.119
SGM populations	983	30.64	2	< 0.001	0.177	982	8.44	2	0.015	0.093
Race/ethnicity	1,001	24.46	8	0.002	0.111	1,000	36.19	8	< 0.001	0.135
Age	1,000	56.13	6	< 0.001	0.168	999	16.23	6	0.013	0.090
Educational level	994	13.59	8	0.093	0.083	993	20.43	8	0.009	0.101
Marital status	991	15.49	6	0.017	0.088	990	9.10	6	0.168	0.068
Annual household income	964	10.52	10	0.396	0.074	963	25.78	10	0.004	0.116
Number of children under 18 in household	1,001	4.95	4	0.293	0.050	1,000	1.43	4	0.840	0.027

Table 5 presents the binary logistic regression among participants providing definitive responses to the experience with mental health illness question (*N* = 895) following the unstable multinomial estimates for the “Not Sure” category for Q2 (i.e., experience with mental illness). Findings revealed that SGM populations, race/ethnicity, and age remained significantly associated with experience with mental illness after adjustment for other sociodemographic characteristics. SGM participants had greater odds of reporting mental illness than heterosexual participants [AOR = 2.29, 95% CI (1.35, 3.97)]. Compared with White respondents, Black/African American [AOR = 0.55, 95% CI (0.37, 0.82)], Hispanic/Latinx [AOR = 0.56, 95% CI (0.38, 0.82)], and Asian respondents [AOR = 0.23, 95% CI (0.11, 0.42)] had lower adjusted odds. Adults aged 45–64 [AOR = 0.55, 95% CI (0.39, 0.79)] and 65 + [AOR = 0.16, 95% CI (0.09, 0.27)] also had lower odds than adults aged 25–44.

**Table 5:** Binary logistic regression of mental illness experience by sociodemographic characteristics (*N* = 895).

Sociodemographic characteristics/contrast	LR χ^2^	df	Omnibus *p*	AOR	95% CI	Contrast *p*
SGM populations	9.61	1	0.002			
SGM vs. heterosexual				**2.29**	**[1.35, 3.97]**	**0.002**
Race/ethnicity	29.08	4	< 0.001			
Black/African American vs. White				**0.55**	**[0.37, 0.82]**	**0.003**
Hispanic/Latinx vs. White				**0.56**	**[0.38, 0.82]**	**0.003**
Asian vs. White				**0.23**	**[0.11, 0.42]**	**< 0.001**
Age	48.95	3	< 0.001			
45–64 vs. 25–44				**0.55**	**[0.39, 0.79]**	**0.001**
65 + vs. 25–44				**0.16**	**[0.09, 0.27]**	**< 0.001**

Outcome, Yes versus No mental illness experience. AOR, adjusted odds ratio. All demographic variables were entered simultaneously. Bolded contrast *p* indicates *p* < 0.05.

Multinomial logistic regression (*N* = 936) showed significant adjusted associations between service awareness and gender, SGM populations, race/ethnicity, age, and annual household income (all *p* < 0.05). Females had greater odds than males of reporting awareness [AOR = 1.56, 95% CI (1.10, 2.20)]. Hispanic/Latinx [AOR = 0.35, 95% CI (0.22, 0.55)] and Asian respondents [AOR = 0.42, 95% CI (0.22, 0.78)] had lower odds of awareness than White respondents. Adults aged 45–64 [AOR = 0.62, 95% CI (0.40, 0.95)] and 65 + [AOR = 0.33, 95% CI (0.18, 0.60)] also had lower odds of awareness than adults aged 25–44. Annual household income and SGM populations were significant overall predictors, although no consistent individual-category effects were observed (Table 6).

**Table 6:** Multinomial logistic regression of awareness of community-based mental health or crisis services by sociodemographic characteristics (*N* = 936).

Sociodemographic characteristics/contrast	LR χ^2^	df	Omnibus *p*	AOR	95% CI	Contrast *p*
Gender	6.85	2	0.033			
Female vs. Male — Yes vs. No				**1.56**	**[1.10, 2.20]**	0.**013**
SGM populations	6.50	2	0.039			
SGM vs. heterosexual — Unsure vs. No				0.94	[0.45, 1.95]	0.861
SGM vs. heterosexual — Yes vs. No				1.84	[0.95, 3.60]	0.072
Race/ethnicity	33.66	8	< 0.001			
Hispanic/Latinx vs. White — Unsure vs. No				**0.56**	**[0.35, 0.89]**	0.**014**
Hispanic/Latinx vs. White — Yes vs. No				**0.35**	**[0.22, 0.55]**	**< 0.001**
Asian vs. White — Yes vs. No				**0.42**	**[0.22, 0.78]**	0.**006**
Age	17.25	6	0.008			
45–64 vs. 25–44 — Unsure vs. No				**0.54**	**[0.35, 0.85]**	0.**008**
65 + vs. 25–44 — Unsure vs. No				**0.48**	**[0.27, 0.87]**	0.**015**
45–64 vs. 25–44 — Yes vs. No				**0.62**	**[0.40, 0.95]**	0.**027**
65 + vs. 25–44 — Yes vs. No				**0.33**	**[0.18, 0.60]**	**< 0.001**
Education level	11.71	8	0.165	—	—	—
Marital status	7.16	6	0.307	—	—	—
Annual household income	18.55	10	0.046			
$100,000–$149,999 — Yes vs. No				**2.01**	**[1.04, 3.91]**	0.**039**
Number of children under 18 in household	0.94	4	0.919	—	—	—

*N* = 936 complete cases. “No” was the reference outcome; comparisons are Unsure vs No and Yes vs No. AOR, Adjusted odds ratio; CI, Confidence interval; LR, Likelihood-ratio test. Bolded contrasts indicate *p* < 0.05.

## Discussion and conclusion

Gender, SGM populations, race/ethnicity, age, and household income remained significantly associated with service awareness after adjustment. Hispanic/Latinx, Asian, older, and male respondents showed lower awareness in significant adjusted contrasts, while SGM populations and annual household income showed significant overall associations.

To begin, older adults showed consistently lower adjusted odds of both reporting mental illness and awareness of community-based mental health or crisis services. Adults aged 65 + had substantially lower odds of reporting mental illness than adults aged 25–44 and also had lower odds of reporting awareness of available services. Previous research suggests that perceived need and mental health literacy may influence recognition and service use among older adults (10–12). These findings suggest continued need for age-responsive mental health literacy and outreach efforts.

SGM population remained independently associated with reported mental illness, with SGM participants having greater adjusted odds of reporting mental illness than heterosexual participants. SGM participants were also significantly associated with overall service awareness, although no individual adjusted awareness contrast reached statistical significance. Prior studies similarly show elevated mental health needs among SGM populations and emphasize the role of supportive, affirming services in help-seeking (6, 13). In contrast, Cronin et al. (14) found that among SGM populations, stress predicted increased barriers to service use, which in turn contributed to poorer mental health outcomes. Awareness, however, should not be interpreted as equivalent to actual service utilization.

Race/ethnicity remained significantly associated with both reported experience with mental illness and awareness of community-based health crisis or services after adjustment. Compared with White respondents, Black/African American, Hispanic/Latinx, and Asian respondents had lower adjusted odds of reporting mental illness, with the largest difference observed among Asian participants. Hispanic/Latinx and Asian respondents also had lower adjusted odds of reporting awareness of available services. These patterns are consistent with literature describing racial and ethnic differences in mental health recognition, stigma, culturally responsive care, and access to services (15–17). These findings should not be interpreted as evidence of lower underlying mental health need within these populations. Differences in recognition, reporting, diagnosis, cultural perceptions, and access to healthcare may contribute to observed disparities in self-reported mental illness and awareness. Number of children under 18 in the household was not independently associated with either reported mental illness or services awareness after adjustment for other sociodemographic characteristics.

Further investigation is needed to understand the relationships among mental illness experience, sociodemographic characteristics, and awareness of community-based services. Although multivariable models accounted for measured sociodemographic characteristics, residual and unmeasured confounding may remain. Both survey waves used non-probability, opt-in samples, and pooling respondents across 2024 and 2026 limits generalizability beyond the respondent group. Self-reported measures, cross-sectional data, differing sampling approaches across survey waves, and sparse responses in selected categories also limit interpretation. Future studies should incorporate longitudinal designs, validated clinical measures, and additional determinants of service awareness and utilization.

In the meantime, MHAGH should continue to raise awareness of community-based mental health or crisis services among adults 65+, Asian, Black/African American, and Hispanic/Latinx participants, as well as SGM populations in the Houston area. Community-based mental health literacy (MHL) programs improve recognition of mental illness (18). Mental health advocates in the Houston area should prioritize MHL campaigns that strengthen mental illness recognition, reporting, and service utilization across the community.

Collaborative efforts between schools, neighborhood organizations, and community leaders may help extend the reach of mental health information. Public healthcare communicators should tailor mental health and crisis service messages to neighborhoods with less awareness, using narratives that normalize help-seeking behaviors and highlight success stories with mental healthcare services. These approaches can help reduce barriers, enhance recognition of mental health needs, and improve equitable access to available services. Finally, future studies should focus on identifying factors contributing to low awareness of mental healthcare services. Identifying these factors can inform outreach strategies and address gaps in mental health awareness.

## Data Availability

The raw data supporting the conclusions of this article will be made available by the authors, without undue reservation.
